# Wheat litter and feed with aluminosilicates for improved growth and meat quality in broiler chickens

**DOI:** 10.7717/peerj.11918

**Published:** 2021-08-04

**Authors:** Mirosław Banaszak, Jakub Biesek, Marek Adamski

**Affiliations:** Department of Animal Breeding and Nutrition, Faculty of Animal Breeding and Biology, UTP –University of Science and Technology in Bydgoszcz, Bydgoszcz, Poland

**Keywords:** Wheat litter, Zeolite, Halloysite, Broiler chicken, Body weight gain, Breast muscles, Raw material quality

## Abstract

**Background:**

Natural minerals have many beneficial properties in poultry production, taking into account production as well as hygiene, health, safety, and quality of broiler meat. The aim of the study was to evaluate the effect of aluminosilicates in feed and litter on the growth performance and meat quality in chickens. Aluminosilicates, including halloysite and zeolite, could be a good alternative for synthetics, as a good solution for the environment in line with the current trends.

**Methods:**

Five-hundred male Ross 308 chickens were managed in 5 groups (10 replicates/group): LITTER: 1, control; 2, 0.95 kg/m^2^ of halloysite; 3, 0.475 kg/m^2^ of halloysite and 0.475 zeolite; 4, 0.95 kg/m^2^ of zeolite; 5, 0.25 kg/m^2^of halloysite and 0.7 kg/m^2^ of zeolite. FEED: groups 2–5, halloysite and zeolite addition (25:75 ratio; 0.5–2%). Growth performance (body weight and feed indicators), carcass, and meat quality (pH, colour, water-holding capacity, chemical composition of muscles) were recorded. The experimental setup, where the aluminosilicate additives were applied simultaneously, was proposed and approved by experts after pilot testing and on the basis of extensive literature where feed or litter additives were tested.

**Results:**

Body weight and its gain were higher in groups 3 and 4 than in 1, and feed intake was higher in 4. The weight of the carcass and some of its components, including muscles and skin with subcutaneous fat, were higher in 2–4. Water loss from leg muscles was lower in 4. The content of protein in muscles was significantly higher in 3. The addition of aluminosilicates in feed and litter had a positive effect on the growth performance and some traits of carcasses and meat quality, especially in group 3. Halloysite and zeolite can be used in feed and litter (especially 0.475 kg/m^2^ for each mineral in the wheat litter).

## Introduction

Consumers need a product with approved high quality, which actually is including of limited influence of food production on natural environment. The solution needs to be improve on beginning of food chain even on the rearing stage of broiler chicken (Petrescu, Vermeir & Petrescu-Mag, 2020; Rosa da et al., 2021).

One of the most popular methods is the use of using antibiotics for keeping the good health status of chickens. The continuous use of antibiotics in poultry has been linked to antimicrobial resistance and drug residue problems (Muaz et al., 2018). Antibiotics, despite the fact that they are intended to eliminate pathogens in poultry, make consumers cautious about poultry meat because residues of antibiotics in poultry products might cause allergic reactions in humans (Lipińska, 2020). Therefore, the question arises of what can be done to rear broiler chickens or other poultry species to achieve good growth performance and quality of meat without antibiotics?

The growth of chickens depends on many factors; however, the greatest attention is paid to genotype and nutrition, including the use of various types of zootechnical additives (Zubair & Leeson, 1996). Relationships between factors should be taken into account, because as a result of the selection of broiler poultry, despite good performance characteristics, there is higher sensitivity to the conditions of keeping and feeding (Skomorucha, Sosnówka-Czajka & Muchacka, 2011).

The high needs of modern broiler chicken breeds determined modification of parts in rearing methods, such as keeping good quality, litter and welfare status together with feed quality, can build effectiveness of production parameters and high quality of meat. It is important for consumers due to the fact that poultry meat is the most common and low cost animal protein. The world’s trends in animal production determine the reduction of the impact on the natural environment. Those recommendations are included in the Green Deal strategy (Karasu & Ozturk, 2020; Peeters et al., 2020; Meda et al., 2021).

There is an ongoing search for alternative solutions that can support poultry production through the use of natural substances stimulating immunity in birds. Such substances include probiotics, *i.e.,* bacterial cultures that have a beneficial effect on the development of the immune system through the digestive system, or prebiotics, symbiotics, organic acids, and enzymes (Alagawany et al., 2018; Wan, Forsythe & El-Nezami, 2019; Montoro-Dasi et al., 2020). To maintain the welfare of birds, substances which are referred to by the authors cited above as “eco-friendly” should, in addition to a positive effect on the avian immune system, also absorb toxic substances (Abd El-Hack et al., 2018) and improve the biosecurity of the livestock facility as well as the performance parameters of broiler chickens (Wina, 2017). Chen et al. (2019) described many natural substances (especially of plant origin) that can replace antibiotics and ensure good growth performance and meat quality in broiler chickens.

Used of mentioned methods are mostly focused on gastrointestinal tract. Very important influence on growth performance and carcass quality has an litter. The type and origin of the litter used in poultry production should also be considered, as indicated in studies by Benabdeljelil & Ayachi (1996) or Bolan et al. (2010). The type of litter used depends on the availability of the material and the country where the birds are kept. These can be materials made of chopped wheat straw, wood shavings, rice husks, dried leaves or coffee husks (Monira et al., 2003). One of the more commonly used bedding materials is wheat litter (Farghly et al., 2021). According to reports, each type of bedding has advantages and disadvantages. The main objective of the research on the quality of litter is its humidity, degree of purity or nitrogen and ammonia content, and the research concerned the use of many additives, such as aluminium sulphate, sodium bisulphate, microbiological preparation or a commercial ammonia binding agent, as well as zeolite. A positive effect has been reported, including minimization of *footpad dermatitis* (Zikic et al., 2017). Referring to the cited literature, the quality of litter and additives improving its condition should be used throughout the production cycle.

Meat quality depends on a number of features defined by physicochemical parameters which indicate the suitability of meat for further processing and consumption (Fletcher, 2002). Factors affecting the quality of meat can be divided into those related to the origin and condition of the birds, *e.g.*, the species, breed, muscle type, sex, age, as well as environmental ones that are related to the management of chickens, *i.e.,* the conditions of keeping and slaughtering, feeding, technological procedures during rearing, and handling the raw material after slaughter and biochemical changes occurring tissues (Tougan et al., 2013).

There is interest in the use of aluminosilicates, which are natural minerals with adsorptive properties and produce versatile pharmacological effects (Semenenko et al., 2020). Among the most popular examples of these minerals are zeolites (or halloysite) used as feed additives, which stimulate the growth of birds and the intestinal morphology, adsorb ammonia, and reduce the incidence of diarrhoea, and even the durability of feeds (Tang et al., 2018). Aluminosilicates used in wheat straw litter have an indirect effect on the level of biosecurity in poultry production (Banaszak et al., 2020). The above-mentioned publications describe the effects of aluminosilicates on growth performance, immune system, organ development, and intestinal morphology, but the literature data on the quality of poultry meat are scarce. In a pilot study on aluminosilicates, Banaszak et al. (2020) found that the use of zeolite was associated with good quality of meat, while halloysite improved the histomorphometric features of the intestines. These researchers suggested the need for further studies in order to determine the appropriate doses of natural supplements in feed and litter.

In connection with the above, it is concluded that poultry meat at the stage of its production and during the rearing of live birds cannot be exposed to factors that may adversely affect the health of birds, humans and the natural environment. The wide use and properties of natural minerals (aluminosilicates) can be an effective and cheap solution in poultry production, and above all, it can be an alternative to synthetic substances used as zootechnical additives in feed and bedding. The undertaken research works described in this paper are fully justified and constitute a specific response to the needs of producers of broiler chicken. The aim of the study was to evaluate and compare growth performance parameters, carcass traits, and meat quality defined by physicochemical parameters in broiler chickens reared with the addition of aluminosilicates in wheat straw litter on the various levels and to the feed-in constant ratio. The research issues undertaken in this paper are aimed at determining the appropriate amount of using aluminosilicates for wheat straw litter, while enhancing their effect as a feed additive.

## Materials & Methods

### Overview of expermiental program

**Figure 1:**
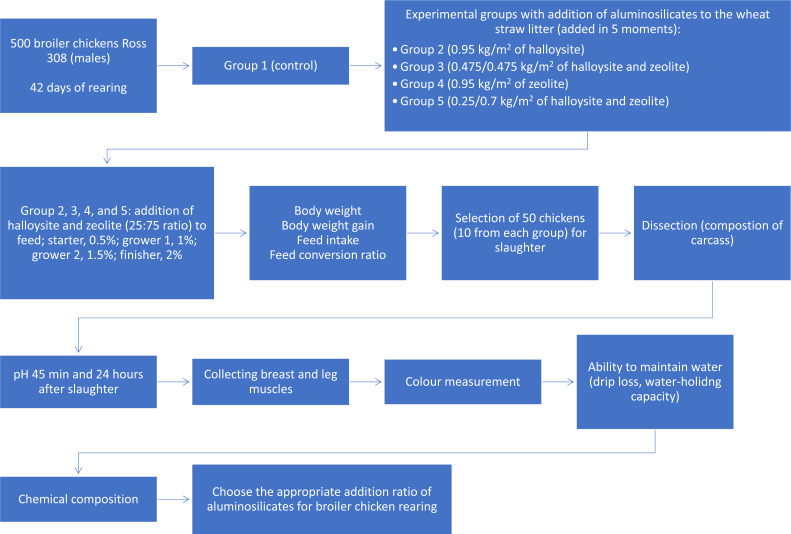
Experimental design and procedures.

This paper aims to present the results of research related to the production of broiler chickens, where a natural feed and litter additive in the form of aluminosilicates (zeolite and halloysite) are used. The control studies covered weight gain and feed consumption indicators, and ultimately the study was aimed at determining the quality of meat from broiler chickens kept with the aforementioned addition of minerals. We divided 500 broiler chickens into five equal groups with 10 replications each. In the feed, the chickens received an addition of halloysite and zeolite (25:75 ratio) at the level of 0.5–2% (groups 2, 3, 4, 5), and different levels of additives were used in the litter (2, 0.95 kg/m^2^ of halloysite; 3, 0.475/0.475 kg/m^2^ of halloysite and zeolite; 4, 0.95 kg/m^2^ of zeolite, and 5, 0.25/0.7 kg/m^2^ of halloysite and zeolite). After 42 days of rearing, the 50 chickens were selected for slaughter and the carcasses were designated for quality analyzes, taking into account the composition of the carcasses as well as the physicochemical parameters of the meat (pH, colour, ability to maintain water, and chemical composition) (Fig. 1).

 The experiment was an R+D project in cooperation with producers of broiler chickens (a form of application research). The birds were managed in commercial conditions, and consistent with Directive no. 2010/63/EU and Decision no. 13/2016 of the Local Ethics Committee of 17 June 2016, it did not require approval from a Local Ethics Committee.

The research is part of the Safe Farm project and a response to the market need related to the use of innovative and natural additives for litter and feed. The experimental setup, where the aluminosilicate additives were applied simultaneously, was proposed and approved by experts after pilot testing and on the basis of extensive literature where feed or litter additives were tested.

### Chicken rearing

The experiment was carried out with five groups. The individual groups of birds in the same environmental conditions were kept. The experiment had the nature of research and development works. In each area, there were 10 pens (replicates), with 10 birds per pen (100 birds per group, total = 500) in each group. In the study, we used male Ross 308 broiler chickens (meat-type hybrids). Groups in replicates (pens) were set up in one building, and thanks to the use of pens, it was possible to fully control the presented experiment and record data. Bird management and diet were consistent with the technology for broiler chicken production (Aviagen, 2015). Birds received commercially available complete feeds. There were four feeding phases: starter (days 1–10), grower 1 (days 11–22), grower 2 (days 23–35), and finisher (days 36–42). In groups, 2–5 each type of feed contained halloysite and zeolite in a 25:75 ratio: 0.5% in starter, 1% in grower 1, 1.5% in grower 2, and 2% in finisher. The feed was granulated. Birds received feed and fresh drinking water *ad libitum*. Halloysite and zeolite were also added to the litter. Data on the allocation to groups and the amount of aluminosilicates added to litter are presented in Table 1. Aluminosilicates were spread on litter 5 times. The chemical composition of aluminosilicates used in the experiment was presented in Table 2. This presented data has been demonstrated on the basis of information obtained from the product supplier. The minerals were in a loose, dusty form. Basic chemical components of feed are presented in Table 3. Chickens were reared for 42 days. The experimental setup, where aluminosilicates were tested by two routes of administration simultaneously, is endorsed by experts and the project consortium and was developed for the needs of a pilot study.

**Table 1:** Description of groups used in the experiment.

Group no.	Number of birds	Replicates (pen × birds)	Addition to litter^a^ (kg/m^2^)	Addition to feed (h:z ratio)	Total use H/Z (kg)
1	100	10 × 10	None	None	None
2	100	10 × 10	0.95 h^1^	starter, 0.5%; grower 1, 1%; grower 2, 1.5%; finisher, 2% (25:75)	78/234
3	100	10 × 10	0.475 h / 0.475 z^2^	starter, 0.5%; grower 1, 1%; grower 2, 1.5%; finisher, 2% (25:75)	78/234
4	100	10 × 10	0.95 z	starter, 0.5%; grower 1, 1%; grower 2, 1.5%; finisher, 2% (25:75)	78/234
5	100	10 × 10	0.25 h / 0.7 z	starter, 0.5%; grower 1, 1%; grower 2, 1.5%; finisher, 2% (25:75)	78/234

**Notes.**

1 h, halloysite

2 z, zeolite

a aluminosilicates were added on 5 dates: days 1, 10, 20, 30, 31 of rearing.

**Table 2:** Chemical composition of zeolite and halloysite.

Components	Zeolite (%)
SiO_2_ (silicon dioxide)	71.30
Al_2_O_3_ (aluminium oxide)	13.10
CaO (calcium oxide)	5.20
K_2_O (potassium oxide)	3.40
Fe_2_O_3_ (iron (III) oxide)	1.90
MgO (magnesium oxide)	1.20
Na_2_O (sodium oxide)	1.30
TiO_2_ (titanium oxide)	0.30
Si/Al (silicon / aluminium)	5.40
Clinoptilolite	84.00
Cristobalit	8.00
Mica clay	4.00
Plagioclases	3.50
Rutile	0.20
**Components**	**Halloysite (%)**
Al (aluminium)	13.00
Si (silicon)	12.00
Ca (calcium)	0.40
Mg (mangesium)	0.30
Na (sodium)	0.10
K (potassium)	0.08
P (phosphorus)	0.30
Fe (iron)	9.00
Ti (titanium)	1.00
Mn (manganese)	0.20

**Table 3:** Chemical composition of feeds for broiler chickens, four feeding phases.

Constituent (%)	STARTER		GROWER 1		GROWER 2		FINISHER	
	C(1)^a^	E(2–5)^b^	C(1)	E(2–5)	C(1)	E(2–5)	C(1)	E(2–5)
Dry matter	86.75	86.67	87.40	86.68	88.27	87.46	87.14	86.83
Crude ash	4.09	7.28	4.88	7.52	7.43	4.86	7.42	4.93
Crude protein	22.25	21.75	21.51	20.46	18.16	18.65	18.66	18.75
Crude fat	4.85	5.16	6.82	6.27	6.94	7.27	7.00	7.50
Crude fibre	3.60	3.44	3.53	3.43	2.67	2.94	3.10	3.14
Starch	39.79	39.78	39.14	38.90	42.04	39.67	40.88	39.26

**Notes.**

a Control group.

b D(2–5), experimental groups; feeds were iso-caloric and iso-protein.

### Growth performance

Broiler chickens were weighed on five dates: day 1 (stocking, starter feed), day 10 (change to grower 1), day 22 (change to grower 2), day 35 (change to finisher), and day 42 (slaughter). Feed intake (FI) was recorded daily, and records were used for the calculation of mean body weight gain (BWG) and feed conversion ratio (FCR).

### Carcass traits and quality

#### Slaughter procedure

After 42 days, 10 birds from each group were randomly chosen for slaughter. Each bird was marked with a jiffy wing band and an ID number. Bands were placed on the right wing of each selected bird. Selected broiler chickens were weighed, starved for 12 h, and slaughtered in accordance with standards and recommendations for the slaughter of birds. The birds were slaughtered by qualified workers by decapitation at the atlanto-occipital joint (rapid exsanguination). Before decapitation, the birds were stunned by an electric current.

The carcasses were scalded in a 60–65 °C water bath and then plucked. Feet were cut off at the ankle joints, carcasses were gutted, and edible offal (heart, gizzard, liver) separated.

#### pH and dissection

The pH value of breast muscles was measured 45 min post-mortem (pH_45min_; *m*. *pectoralis major)* using a pH-meter (Elmetron, Zabrze, Poland) with a knife electrode. The carcasses were chilled for 24 h in a cold room (Hendi, Poznań, Poland) at 4 °C, weighed together with offal (Radwag, Radom, Poland), and measured for pH again (pH_24hours_). Carcasses were dissected by separating the neck with skin, wings with skin, skin with subcutaneous fat, abdominal fat, breast muscles, leg muscles (thighs and drumsticks; without bones) and carcass remains (body, leg bones). Each carcass element was weighed. Breast muscles (right and left) and leg muscles (right and left) were kept for further laboratory analyses.

#### Colour

Right breast and leg muscles were analysed for colour in the CIE Lab system, including lightness (L*), redness (a*), and yellowness (b*). Measurements were taken with a colorimeter (Konica Minolta, Tokyo, Japan) on the outer side of the muscle (Traffano-Schiffo et al., 2021).

#### Drip loss

After colour assessment right breast muscles were weighed (M1), placed in perforated plastic bags with zip closure (to enable spontaneous dripping of liquid), and packed in larger bags with a zip closure to collect liquid. Samples were left to hang in a cold room at 4 °C for 24 h, weighed again (M2), and the drip loss from breast muscles was calculated in percent.

#### Water holding capacity

Left breast and leg muscles were disintegrated in a mincer (Hendi, Poznań, Poland). Five samples of minced meat from each group were prepared (0.300 g, ±0.005 g) (M1), placed between two pieces of Whatman 1 filter paper, and kept under 2 kg pressure for 5 min. After that time samples were weighed again (M2), and the loss of water (water holding capacity) was calculated in percent.

pH, drip loss, and water holding capacity were calculated using formulas described by Zhuang et al. (2020).

#### Chemical composition

Samples of minced breast and leg muscles (90 g) were analysed for chemical composition (the content of protein, collagen, sodium chloride, intramuscular fat, and water) in 5 replicates for each group. Analyses were done using near-infrared transmission (NIT) spectrometry (FoodScan, FOSS, Hilleroed, Denmark). The analysis of carcass traits and physicochemical traits of meat followed methods described by Biesek et al. (2020).

### Statistical analysis

Numerical data were processed with Statistica software 13.0 (2017, Statsoft, Kraków, Poland). The statistical model of one-way analysis of variance (ANOVA) was used, in relation to the grouping factor (addition of aluminosilicates). Means for each analysed trait in groups and the standard error of the mean (SEM) were calculated using the subclass effect model. Statistically significant differences were verified using the post-hoc test (Sheffe’s test), at the significance level *p* < 0.05. Additionally, F-values and adjusted coefficient of determination (adj. R^2^) were calculated (steps in statistical software: GLM, generalized linear model; intergroup effects, full model R, alpha values: confidence, 0.950; significance, 0.050, full model SS test *versus* residual SS test). F-value calculations were based on the components: SS, intra-sample variability, and intergroup variability (sum of squares); df, number of degrees of freedom; MS, mean square of variation between and within groups. The variance of the population of all groups was homogeneous. The variance of the population of all groups was homogeneous. The distribution of the results of the dependent variables in each of the analysed groups was close to the normal distribution. In accordance with the principles of experiments, samples for qualitative analysis were taken randomly, which indicates that project randomization was used.

## Results

The mortality of birds did not exceed 1% and concerning disabled and weak chicks at the early stage of rearing.

### Growth performance

Data on growth performance are presented in Table 4. The chicks in all groups on the day of stocking had similar body weight (*p* = 0.059, *F* = 2.97, R^2^(adj.) = 0.14). On days 22, 35 and 42, the body weight of broiler chickens was significantly lowest in groups 1 and 5 (0.25 kg/m^2^ of zeolite and 0.7 kg/m^2^ of halloysite in litter) (*p* < 0.001, *F* = 11.40, 17.65, 35.53, R^2^(adj.) = 0.46, 0.58, 0.74, respectively). Body weight gain (BWG) was significantly higher in groups 2 (0.95 kg/m^2^ of halloysite in the litter), 3 (0.475 kg/m^2^ of halloysite and 0.475 kg/m^2^ of zeolite in the litter), and 4 (0.95 kg/m^2^ of zeolite in the litter) in the second feeding phase when birds were on the starter diet (days 10–21) (*p* < 0.001, *F* = 10.15, R^2^(adj.) = 0.43). In the third feeding phase (days 23–35) BWG was significantly higher in all of experimental groups than in group 1 (*p* < 0.001, *F* = 7.68, R^2^(adj.) = 0.35), while in the whole rearing period (days 1–42) it was significantly higher in groups 3 and 4 (p < 0.001), *F* = 35.48, R^2^(adj.) = 0.74). Similar relationships were found for feed intake (FI), which was significantly lowest in groups 1 and 5 (phase 3: days 23–35) (*p* < 0.001), *F* = 18.04, R^2^(adj.) = 0.58), groups 1 and 2 (phase 4: days 36–42) (*p* < 0.001, *F* = 16.67, R^2^(adj.) = 0.32), and for the whole rearing period FI was significantly lowest in groups 1, 2 and 5 (*p* < 0.001, *F* = 6.68, R^2^(adj.) = 0.32). Feed conversion ratio (FCR) differed significantly between groups during the second feeding phase and was higher in groups 1, 4, and 5 (*p* < 0.001, *F* = 7.49, R ^2^(adj.) = 0.35).

**Table 4:** Growth performance of broiler chickens during the 42 days of rearing.

Parameter^2^ *n=100 per group*	Group^1^					SEM	*p*-value	*F*-value	R^2^(adj.)
	1	2	3	4	5				
BW (g)									
1-day-old chicks	36.48	35.68	36.32	36.67	36.76	0.12	0.059	2.97	0.14
day 10	329.09	337.73	345.45	345.27	331.02	2.41	0.079	2.25	0.09
day 22	1015.32^c^	1107.07^b^	1138.98^a^	1102.85^b^	1020.12^c^	10.05	<0.001	11.40	0.46
day 35	2147.18^c^	2411.07^ab^	2496.17^ab^	2501.10^a^	2342.00^b^	23.80	<0.001	17.65	0.58
day 42	2604.03^d^	2917.02^bc^	3061.48^a^	3041.89^ab^	2893.07^c^	26.84	<0.001	35.53	0.74
BWG (g)									
days 1–10	292.61	302.05	309.13	308.60	294.26	2.38	0.069	2.35	0.10
days 11–22	686.23^c^	769.34^a^	793.53^a^	757.58^ab^	689.09^bc^	9.05	<0.001	10.15	0.43
days 23–35	1131.86^b^	1304.00^a^	1357.19^a^	1398.25^a^	1321.88^a^	20.47	<0.001	7.68	0.35
days 36–42	456.86	505.96	565.32	540.79	551.07	16.62	0.245	1.41	0.13
days 1–42	2567.55^d^	2881.34^bc^	3025.16^a^	3005.22^ab^	2856.31^c^	26.85	<0.001	35.48	0.74
FI (g; per bird)									
days 1–10	315.74	321.90	339.64	324.77	332.87	3.04	0.101	2.07	0.08
days 11–22	1011.11	1022.22	1011.11	1033.33	1011.11	5.82	0.691	0.56	0.14
days 23–35	1881.83^c^	2276.20^a^	2184.76^ab^	2313.99^a^	1997.76^bc^	30.12	<0.001	18.04	0.58
days 36–42	844.89^b^	868.51^b^	1097.82^a^	1098.51^a^	1011.62^a^	20.17	<0.001	16.67	0.56
days 1–42	4318.06^d^	4539.73^abcd^	4773.13^bc^	4971.71^ab^	4466.68^cd^	54.19	<0.001	6.68	0.32
FCR (kg/kg)									
days 1–10	1.08	1.07	1.10	1.05	1.14	0.01	0.149	1.78	0.06
days 11–22	1.48^a^	1.33^bc^	1.27^c^	1.38^abc^	1.47^ab^	0.02	<0.001	7.49	0.35
days 23–35	1.66	1.75	1.61	1.66	1.54	0.03	0.102	2.06	0.08
days 36–42	1.87	1.79	2.06	2.12	1.95	0.07	0.591	0.71	0.10
days 1–42	1.68	1.58	1.58	1.66	1.56	0.01	0.058	3.01	0.14

**Notes.**

a, b, c, d, means in the same line with no common superscript differ between groups (*p* < 0.05); SEM, standard error of the mean; R^2^(adj.), adjusted coefficient of determination.

1 1, no addition of aluminosilicates to feed and litter; 2, 0.95 kg/m^2^ of halloysite in litter; 3, 0.475 kg/m^2^ of halloysite and 0.475 kg/m^2^ of zeolite in litter; 4, 0.95 kg/m^2^ of zeolite in litter; 5, 0.25 kg/m^2^ of halloysite and 0.7 kg/m^2^ of zeolite in litter; groups 2–4, addition of halloysite and zeolite in feed (proportion 25:75; starter, 0.5%; grower 1, 1%; grower 2, 1.5%; finisher, 2%).

2 BW, body weight, g; BWG, body weight gain, g; FI, feed intake, g; FCR, feed conversion ratio, kg/kg.

### Carcass traits

The analysis of data on the traits of broiler chicken carcass (Table 5) from birds selected for slaughter revealed a significantly lower body weight (*p* < 0.001, *F* = 21.86, R^2^(adj.) = 0.70) and carcass weight (*p* < 0.001, *F* = 33.46, R^2^(adj.) = 0.79) in group 1 compared to other experimental groups 2–5. However, dressing percentage did not differ significantly between the groups and was in the range of 74.06–77.84% (*p* = 0.059, *F* = 9.28, R^2^(adj.) = 0.13). The weight of the neck with skin was highest in groups 3–5 (*p* < 0.001, *F* = 18.08, R^2^(adj.) = 0.38), and the proportion of the neck with skin in the carcass was highest in group 1 (*p* = 0.011, *F* = 1.84, R^2^(adj.) = 0.18). The weight of wings was significantly higher in experimental groups (2–5) than in the control group (1) (*p* < 0.001, *F* = 12.47, R^2^(adj.) = 0.50), and the proportion of wings in carcass was significantly higher in group 1 than in group 3 (*p* −0.002, *F* = 9.19, R^2^(adj.) = 0.25). Considering the weight of offal, the analysis revealed a significantly lower weight of the heart in group 1 compared to groups 2–3 (*p* < 0.001, *F* = 5.92, R^2^(adj.) = 0.35), while the weight of carcass remains was significantly lower in group 1 than in all experimental groups (2–5) (*p* < 0.001, *F* = 9.27, R^2^(adj.) = 0.34).

**Table 5:** Traits of broiler chicken carcass Parameter *n* = 10 per group.

	Group^1^					SEM	*p*-value	*F*-value	R^2^(adj.)
	1	2	3	4	5				
Pre-slaughter body weight (g)	2574.00^b^	3098.00^a^	3230.00^a^	3178.00^a^	3080.00^a^	39.44	<0.001	21.86	0.70
Weight of carcass (g)	1899.81^b^	2357.41^a^	2485.67^a^	2471.34^a^	2388.07^a^	34.37	<0.001	33.46	0.79
Dressing percentage (%)	74.06	76.08	76.95	77.84	77.54	0.44	0.059	9.28	0.13
Neck with skin (g)	101.11^c^	106.05^bc^	129.82^ab^	140.12^a^	125.71^abc^	3.22	<0.001	18.08	0.38
Neck with skin (%)	5.34^ab^	4.49^b^	5.21^ab^	5.67^a^	5.28^ab^	0.11	0.011	1.84	0.18
Wings (g)	185.57^b^	223.28^a^	217.07^a^	224.46^a^	216.05^a^	2.76	<0.001	12.47	0.50
Wings (%)	9.78^a^	9.48^ab^	8.74^b^	9.09^ab^	9.05^ab^	0.09	0.002	9.19	0.25
Heart (g)	9.66^b^	12.22^a^	13.10^a^	12.95^a^	11.59^ab^	0.28	<0.001	5.92	0.35
Gizzard (g)	28.19	29.30	29.92	29.04	28.66	0.57	0.909	0.02	0.07
Liver (g)	55.72	68.01	64.49	66.89	62.52	1.54	0.085	1.33	0.09
Carcass remains (g)	485.56^b^	624.12^a^	616.50^a^	609.00^a^	611.25^a^	11.86	<0.001	9.27	0.34

**Notes.**

a, b, c, d, means in the same line with no common superscript differ between groups (*p* < 0.05); SEM, standard error of the mean; R^2^ (adj.), adjusted coefficient of determination.

1 1, no addition of aluminosilicates to feed and litter; 2, 0.95 kg/m^2^ of halloysite in litter; 3, 0.475 kg/m^2^ of halloysite and 0.475 kg/m^2^ of zeolite in litter; 4, 0.95 kg/m^2^ of zeolite in litter; 5, 0.25 kg/m^2^ of halloysite and 0.7 kg/m^2^ of zeolite in litter; groups 2-4, addition of halloysite and zeolite in feed (proportion 25:75; starter, 0.5%; grower 1, 1%; grower 2, 1.5%; finisher, 2%).

Table 6 presents data on the weight and proportion of muscles and fat in broiler chicken carcass. The weight of breast muscles was significantly lower in group 1 compared to groups 3–5, and in group 2 compared to group 3 (*p* < 0.001, *F* = 32.47, R^2^(adj.) = 0.72). The proportion of breast muscles in carcass was also significantly lower in group 1 than in group 3 (*p* = 0.016, *F* = 3.38, R^2^(adj.) = 0.48). In group 1 significantly lower the weight of leg muscles (*p* < 0.001, *F* = 13.19, R^2^(adj.) = 0.50), the total weight of muscles (*p* < 0.001, *F* = 30.82, R^2^(adj.) = 0.72), and the total weight of fat (*p* < 0.001, *F* = 0.67, R^2^(adj.) = 0.06) than in other groups (2–5). The weight of skin with subcutaneous fat was also significantly lower in group 1 than in groups 3–5 (*p* < 0.001, *F* = 9.53, R^2^(adj.) = 0.41). There were no significant differences between other analysed traits (*p* > 0.05).

**Table 6:** Content of muscles and fat in broiler chicken carcass.

Parameter *n* = 10 per group	Group^1^					SEM	*p*-value	*F*-value	R^2^(adj.)
	1	2	3	4	5				
Weight and proportion in carcass									
Breast muscles (g)	559.35^c^	710.66^b^	797.29^a^	771.22^ab^	727.81^ab^	13.74	< 0.001	32.47	0.72
Breast muscles (%)	29.44^b^	30.16^ab^	32.08^a^	31.22^ab^	30.47^ab^	0.27	0.016	3.38	0.48
Leg muscles (g)	425.55^b^	519.73^a^	547.70^a^	550.05^a^	525.20^a^	8.88	<0.001	13.19	0.50
Leg muscles (%)	22.41	22.00	22.04	22.22	22.02	0.19	0.962	0.15	0.11
Total muscles (g)	984.90^b^	1230.39^a^	1344.99^a^	1321.27^a^	1253.01^a^	21.39	< 0.001	30.82	0.72
Total muscles (%)	51.85	52.16	54.12	53.44	52.48	0.33	0.153	1.77	0.36
Skin with subcutaneous fat (g)	167.43^b^	196.20^ab^	214.84^a^	215.68^a^	217.29^a^	4.02	<0.001	9.53	0.41
Skin with subcutaneous fat (%)	8.84	8.33	8.63	8.72	9.11	0.13	0.394	1.05	0.09
Abdominal fat (g)	17.17	23.82	18.93	20.79	20.48	1.07	0.388	1.06	0.09
Abdominal fat (%)	0.91	1.00	0.76	0.85	0.86	0.05	0.593	0.71	0.06
Total fat (g)	184.60^b^	220.02^a^	233.77^a^	236.47^a^	237.77^a^	4.34	<0.001	8.56	0.38
Total fat (%)	9.75	9.33	9.39	9.57	9.97	0.14	0.618	0.67	0.06

**Notes.**

a, b, c, d, means in the same line with no common superscript differ between groups (*p* < 0.05); SEM, standard error of the mean; R^2^(adj.), adjusted coefficient of determination.

1 1 , no addition of aluminosilicates to feed and litter; 2, 0.95 kg/m^2^ of halloysite in litter; 3, 0,475 kg/m^2^ of halloysite and 0,475 kg/m^2^ of zeolite in litter; 4, 0,95 kg/m^2^ of zeolite in litter; 5, 0,25 kg/m^2^ of halloysite and 0,7 kg/m^2^ of zeolite in litter; groups 2–4, addition of halloysite and zeolite do feed (proportion 25:75; starter, 0.5%; grower 1, 1%; grower 2, 1.5%; finisher, 2%).

### Physicochemical traits of breast and leg muscles

The analysis of breast muscles revealed that pH 45 min post-mortem (pH_45min_) was significantly higher in group 2 than in group 4 (*p* = 0.016, *F* = 3.41, R^2^(adj.) = 0.16). The water-holding capacity of leg muscles was significantly lowest (highest loss of water) in group 5 compared to group 4 (*p* = 0.012, *F* = 3.61, R^2^(adj.) = 0.18). The content of protein in breast and leg muscles was significantly higher in group 3 compared to other groups, while the content of intramuscular fat in group 3 was lowest in leg muscles, and in group 2 in breast muscles. The content of collagen was significantly lower in group 3 than in group 2 for breast muscles and in groups 3 and 4 than in group 1 for leg muscles. A high coefficient of determination (R^2^(adj.)) was demonstrated in the protein content at the level of 0.97–0.98, as well as water content −0.98 in the breast and leg muscles, and in the leg muscles, also in the intramuscular fat (R^2^(adj.) = 0.99) with significance level *p* <** 0.001. Other analysed parameters presented in Table 7 did not differ significantly between groups (*p* >0.05).

**Table 7:** Physicochemical parameters of breast and leg muscles from broiler chicken.

Parameter^2^ *n=10 per group*	Group^1^					SEM	*p-* value	*F*-value	R^2^(adj.)
	1	2	3	4	5				
Breast muscles									
pH_45_	6.19^ab^	6.42^a^	6.21^ab^	6.16^b^	6.19^ab^	0.03	0.016	3.41	0.16
pH_24_	5.89	6.00	5.96	5.95	5.96	0.02	0.371	1.09	0.09
Colour									
L*	50.50	49.46	48.07	49.39	50.85	0.43	0.271	1.34	0.03
a*	2.96	2.74	3.45	3.02	2.50	0.19	0.730	0.51	0.04
b*	2.62	3.77	3.09	4.44	3.03	0.29	0.299	1.26	0.02
Water-holding capacity (%)	34.89	32.01	34.38	34.80	34.70	0.37	0.061	2.43	0.10
Drip loss (%)	1.39	0.91	0.97	0.73	0.90	0.07	0.051	2.59	0.12
Protein (%)	22.37^b^	22.00^d^	22.86^a^	22.15^c^	22.35^b^	0.04	< 0.001	2.97	0.97
Collagen (%)	0.74^ab^	0.85^a^	0.64^b^	0.73^ab^	0.81^ab^	0.02	0.007	0.09	0.20
Salt (%)	0.25	0.20	0.21	0.22	0.23	0.01	0.547	0.02	0.06
Intramuscular fat (%)	2.38^a^	1.90^b^	2.27^ab^	2.25^ab^	2.54^a^	0.05	< 0.001	0.08	0.32
Water (%)	74.70^c^	75.62^a^	74.36^d^	75.40^b^	74.62^c^	0.07	< 0.001	3.92	0.98
Leg muscles									
Colour									
L*	51.21	46.42	46.79	46.89	47.35	0.63	0.092	2.14	0.08
a*	7.75	8.02	8.04	8.93	7.41	0.45	0.876	0.30	0.03
b*	5.47	5.27	5.23	4.05	5.19	0.29	0.575	0.73	0.06
Water-holding capacity (%)	35.66^ab^	34.24^ab^	33.67^ab^	33.47^b^	40.67^a^	0.78	0.012	3.61	0.18
Protein (%)	18.78^d^	19.35^c^	20.10^a^	19.61^b^	18.79^d^	0.07	< 0.001	0.29	0.98
Collagen (%)	1.26^a^	1.14^ab^	1.03^b^	1.10^b^	1.11^ab^	0.02	< 0.001	7.71	0.30
Salt (%)	0.48^a^	0.32^b^	0.31^b^	0.36^b^	0.31^b^	0.01	< 0.001	18.16	0.58
Intramuscular fat (%)	5.79^c^	6.53^b^	4.34^*e*^	5.12^d^	6.71^a^	0.13	< 0.001	0.22	0.99
Water (%)	74.17^c^	73.22^*e*^	74.84^a^	74.60^b^	73.41^d^	0.09	< 0.001	726.73	0.98

**Notes.**

a, b, c, d, means in the same line with no common superscript differ between groups (*p* < 0.05); SEM, standard error of the mean; R^2^(adj.), adjusted coefficient of determination.

1 1, no addition of aluminosilicates to feed and litter; 2, 0.95 kg/m ^2^ of halloysite in litter; 3, 0.475 kg/m^2^ of halloysite and 0.475 kg/m^2^ of zeolite in litter; 4, 0.95 kg/m^2^ of zeolite in litter; 5, 0.25 kg/m^2^ of halloysite and 0.7 kg/m^2^ of zeolite in litter; groups 2–4, addition of halloysite and zeolite in feed (proportion 25:75; starter, 0.5%; grower 1, 1%; grower 2, 1.5%; finisher, 2%).

2 L*, lightness; a*, redness; b*, yellowness.

## Discussion

In poultry production, it is important to achieve a high body weight of birds at a low feed conversion ratio per kg of body weight gain. The addition of natural zeolite at a level of 15 and 25 g/kg of feed had no effect on the body weight of broiler chickens or feed intake (Cabuk et al., 2004). Nevertheless, researchers reported a significantly higher FCR compared to chickens on a diet with or without the addition of *Yucca schidigera*. Zhou et al. (2014) demonstrated that a 2% inclusion of zeolite and attapulgite (1:1) in feed improved body weight gain and feed intake, but had no significant effect on FCR, which was also found in our study or in experiments with a 1.5 and 3% inclusion of zeolite (Safaei Katouli et al., 2010) (Refer to Table 4).

Other authors (Tang et al., 2014) investigated the effect of a diet with zinc-bearing zeolite on broiler chickens. They found no significant differences in body weight gain and feed intake between control and experimental birds but reported a lower feed: gain ratio in the group of chickens on a diet with zinc-bearing zeolite clinoptiololite. In our study, the body weight and body weight gain were significantly higher in birds that received 0.5–2% of halloysite with zeolite (1:1) in feed and 0.475 kg/m^2^ of halloysite and 0.475 kg/m ^2^ of zeolite in the litter (group 3) and in the group (4) that received zeolite in feed at a level of 0.95 kg/m^2^ of litter compared to the control group (1). Feed intake was significantly higher in group 4 than in group 1 (Refer to Table 4). Differences between our findings and those reported by cited authors may be due to different doses of zeolite and other substances that were proposed in studies. The higher feed intake indicates that the addition of aluminosilicates may stimulate birds’ appetite.

Schneider et al. (2016) analysed the addition of zeolite in feed (5 g/kg) and litter (100 g/kg). They found no significant effect of zeolite on growth performance or dressing percentage. However, in our study, the body weight and carcass weight were significantly higher in all experimental groups (with aluminosilicates) compared to the control group, but there were no significant differences in dressing percentage between groups (Refer to Table 5), which is consistent with a study investigating the use of 3 and 4.5% of natural zeolite in chicken feed (Khajali, Zamani Moghadam & Habibi, 2006). Differences in results for growth performance and dressing percentage may also be attributed to factors discussed by Schneider et al. (2016), or Al-Nasser et al. (2011), who found improved growth performance in the summer season, but not in winter. The own research did not investigate the influence of other factors, such as the season. The production of broilers should be uniform throughout the year, regardless of the outside temperatures, as the environmental conditions in the buildings of intensive production farms are fully controlled. However, it may be related to the hygiene of litter or manure, which has not been examined in the presented study. In summer, the amount of microorganisms and released gases may be greater because the conditions are more favorable. Also, the period in which the broiler chicks are harvested may be important for the production effectiveness, as it is related to the age of the parent stock.

Khajali, Zamani Moghadam & Habibi (2006) did not observe any significant effects of different zeolite levels in feed on the traits of carcass, breast muscles, or leg muscles. Our research showed a positive effect of aluminosilicates in feed and litter (especially in group 3) on the weight of breast muscles and their proportion in carcass, as well as the weight of leg muscles and muscles in total (Refer to Table 6). The use of 2% of natural zeolite in feed increased the body weight as well as dressing percentage, thus increasing the proportion of muscles in the carcass (Christaki et al., 2001). Our findings also imply that the higher weight of breast muscles is associated with carcass weight (Refer to Table 6). The improved proportion of breast muscles in carcass was also reported by Esmaili et al. (2017), who investigated the effects of zeolite in combination with silver nanoparticles. Yalcin, Bilgili & McDaniel (1995) observed a significant effect of sodium zeolite on the proportion of smaller breast muscles (*pectoralis minor*). In our study, we also found that the use of aluminosilicates in chicken feed was associated with a higher weight of skin with subcutaneous fat and total content of fat in carcass (Refer to Table 6). Hcini et al. (2018) analysed the effect of 2% of zeolite in feed and reported improved growth performance, and better characteristics of breast muscles and meat quality in chicken, but also a positive effect of zeolite on the level of polyunsaturated fatty acids in turkeys. Therefore, the higher content of fat in chicken carcass found in our study should not be regarded as a negative effect considering progress in poultry production and selective breeding (Refer to Table 7). Further studies are needed in this regard to analyse the composition of fatty acids.

In all groups of broiler chicken, the value of pH_24hours_ was at a similar level (5.96–6.00) (Refer to Table 7). The pH value of muscles depends on the post-mortem biochemical changes in muscle fibers (Swatland, 2008), as well as factors potentially inducing pre-slaugther stress in birds (Allen, Russell & Fletcher, 1997), so differences in pH_45min_ between experimental groups could be attributed to an individual response of birds to stressors (Refer to Table 7). According to Ristic & Damme (2010), the standard value of pH for broiler chicken meat without defects is 5.9–6.2. Meat colour assessed in our study (in breast muscles and leg muscles) suggested the absence of the light / dark defect in relation to findings by Fletcher, Qiao & Smith (2000), who reported the mean value of L* = 50.8 for light meat, 47.6 for normal colour meat, and 45.4 for dark meat, and this trait is correlated with pH value. Only in the control group in our study was the lightness of meat (L*) on the borderline between light and normal (Refer to Table 7). The water-holding capacity of meat may be dependent on, and modified by, the oxidation of proteins (Hashemi et al., 2014). In our study the addition of aluminosilicates in feed and litter was associated with a lower loss of water, especially in the group of birds where zeolite was used only in litter (Refer to Table 7). Zhou et al. (2014) suggest that the addition of zeolite can enhance the antioxidant system. Mallek et al. (2012) concluded that there is some interaction between zeolite and muscle proteins that changes the texture and microstructure of muscles, including the gelation of proteins. Our study revealed that the content of protein was significantly higher in breast muscles and leg muscles from chickens where the addition of aluminosilicates was used. It can be concluded that a protein with a larger gel structure loses less water, which was also demonstrated in our research. However, the content of protein was significantly lower in the group where halloysite was also used in the litter (Refer to Table 7). A pilot study by Banaszak et al. (2020) demonstrated a positive effect of zeolite on meat quality parameters, while halloysite had a positive effect on the intestinal morphology associated with improved absorption of nutrients and growth of birds. As indicated by the quoted authors (Schneider et al., 2016; Al-Nasser et al., 2011), the use of different types of aluminosilicates may have benefits in the form of lower production costs. Analysing the production results from our own studies, weight gain and proportional feed consumption did not indicate a very improved efficiency, however, the FCR in the first group was quantitatively higher compared to the experimental groups (only in group 4 it was a slight difference). Nevertheless, statistical verification did not confirm any significant differences (Refer to Table 4). Based on the works in which the requested beneficial effects of aluminosilicates on many elements related to the production (growth performance, hygiene litter, reduced the ammonia levels, the health of the intestine) may assume a beneficial financial aspect since the use of natural minerals reduced by the costs associated with intervention veterinary practice.

## Conclusions

Due to the first hypothesis which was tested, the addition of aluminosilicates in feed and litter had a positive effect on growth performance parameters, carcass traits, and meat quality, as indicated by values measured for many analysed traits. The addition of halloysite and zeolite (25:75) at levels of 0.5–2% in feed for broiler chickens could improve feed assimilability, as shown by higher body weight and body weight gains, as well as the total weight of muscles. Considering the effect of aluminosilicates in the litter, the best results were obtained in the group of birds where 0.475 kg/m^2^of halloysite and zeolite were added in the wheat litter. Values obtained in our study for the weight of muscles, water-holding capacity, and the content of protein in muscles are consistent with many other reports on the correlations between these traits. The inconsistencies that have been found between our study and some cited reports may suggest that the origin of broiler chickens, management conditions and type of litter used should also be considered. Summing up, the research indicates that halloysite and zeolite can be used in feed (25:75 ratio, 0.5–2%) and in the wheat straw litter (0.475 kg/m^2^ for each aluminosilicate), which is a response to the second part of the research hypothesis. The present study is part of a large project aimed at investigating the applicability of aluminosilicates in feed and litter during the production of broiler chickens, and further research is needed due to the various types of litter or even production systems, and their conditions.

##  Supplemental Information

10.7717/peerj.11918/supp-1 Supplemental Information 1 Carcass and meat quality raw data

10.7717/peerj.11918/supp-2 Supplemental Information 2 Raw data of growth performance

10.7717/peerj.11918/supp-3 Supplemental Information 3 Author Checklist

## Additional Information and Declarations

### Data Availability

The following information was supplied regarding data availability: Raw data are available as Supplementary Files.
